# Supplementary material for: A fault-intrusion-tolerant system and deadline-aware algorithm for scheduling scientific workflow in the cloud
Source: PeerJ Comput Sci. 2021 Nov 2;7:e747. doi: 10.7717/peerj-cs.747 (PMC8576554; doi:10.7717/peerj-cs.747)
Supplement: Supplemental Information 6 [file peerj-cs-07-747-s006.zip › Myproject1.1/docs/org/cloudbus/cloudsim/power/PowerVmSelectionPolicyMaximumCorrelation.html]

PowerVmSelectionPolicyMaximumCorrelation


JavaScript is disabled on your browser.


- Overview
- Package
- Class
- Use
- Tree
- Deprecated
- Index
- Help

- Prev Class
- Next Class

- Frames
- No Frames

- All Classes

- Summary:
- Nested |
- Field |
- Constr |
- Method

- Detail:
- Field |
- Constr |
- Method


org.cloudbus.cloudsim.power

## Class PowerVmSelectionPolicyMaximumCorrelation

- java.lang.Object
- - org.cloudbus.cloudsim.power.PowerVmSelectionPolicy
  - - org.cloudbus.cloudsim.power.PowerVmSelectionPolicyMaximumCorrelation

- ---

    

  ```
  public class PowerVmSelectionPolicyMaximumCorrelation
  extends PowerVmSelectionPolicy
  ```

  The Maximum Correlation (MC) VM selection policy.
  If you are using any algorithms, policies or workload included in the power package, please cite
  the following paper:
  Anton Beloglazov, and Rajkumar Buyya, "Optimal Online Deterministic Algorithms and Adaptive
  Heuristics for Energy and Performance Efficient Dynamic Consolidation of Virtual Machines in
  Cloud Data Centers", Concurrency and Computation: Practice and Experience (CCPE), Volume 24,
  Issue 13, Pages: 1397-1420, John Wiley & Sons, Ltd, New York, USA, 2012

  Since:
  :   CloudSim Toolkit 3.0

- - ### Constructor Summary

    Constructors

    Constructor and Description

    `PowerVmSelectionPolicyMaximumCorrelation(PowerVmSelectionPolicy fallbackPolicy)`

    Instantiates a new power vm selection policy maximum correlation.
  - ### Method Summary

    Methods

    | Modifier and Type | Method and Description |
    | --- | --- |
    | `protected java.util.List<java.lang.Double>` | `getCorrelationCoefficients(double[][] data)` Gets the correlation coefficients. |
    | `PowerVmSelectionPolicy` | `getFallbackPolicy()` Gets the fallback policy. |
    | `protected int` | `getMinUtilizationHistorySize(java.util.List<PowerVm> vmList)` Gets the min utilization history size. |
    | `protected double[][]` | `getUtilizationMatrix(java.util.List<PowerVm> vmList)` Gets the utilization matrix. |
    | `Vm` | `getVmToMigrate(PowerHost host)` Gets the vms to migrate. |
    | `void` | `setFallbackPolicy(PowerVmSelectionPolicy fallbackPolicy)` Sets the fallback policy. |

    - ### Methods inherited from class org.cloudbus.cloudsim.power.PowerVmSelectionPolicy

      `getMigratableVms`
    - ### Methods inherited from class java.lang.Object

      `clone, equals, finalize, getClass, hashCode, notify, notifyAll, toString, wait, wait, wait`

- - ### Constructor Detail


    - #### PowerVmSelectionPolicyMaximumCorrelation

      ```
      public PowerVmSelectionPolicyMaximumCorrelation(PowerVmSelectionPolicy fallbackPolicy)
      ```

      Instantiates a new power vm selection policy maximum correlation.

      Parameters:
      :   `fallbackPolicy` - the fallback policy
  - ### Method Detail


    - #### getVmToMigrate

      ```
      public Vm getVmToMigrate(PowerHost host)
      ```

      **Description copied from class: `PowerVmSelectionPolicy`**

      Gets the vms to migrate.

      **Specified by:**
      :   `getVmToMigrate` in class `PowerVmSelectionPolicy`

      Parameters:
      :   `host` - the host

      Returns:
      :   the vms to migrate


    - #### getUtilizationMatrix

      ```
      protected double[][] getUtilizationMatrix(java.util.List<PowerVm> vmList)
      ```

      Gets the utilization matrix.

      Parameters:
      :   `vmList` - the host

      Returns:
      :   the utilization matrix


    - #### getMinUtilizationHistorySize

      ```
      protected int getMinUtilizationHistorySize(java.util.List<PowerVm> vmList)
      ```

      Gets the min utilization history size.

      Parameters:
      :   `vmList` - the vm list

      Returns:
      :   the min utilization history size


    - #### getCorrelationCoefficients

      ```
      protected java.util.List<java.lang.Double> getCorrelationCoefficients(double[][] data)
      ```

      Gets the correlation coefficients.

      Parameters:
      :   `data` - the data

      Returns:
      :   the correlation coefficients


    - #### getFallbackPolicy

      ```
      public PowerVmSelectionPolicy getFallbackPolicy()
      ```

      Gets the fallback policy.

      Returns:
      :   the fallback policy


    - #### setFallbackPolicy

      ```
      public void setFallbackPolicy(PowerVmSelectionPolicy fallbackPolicy)
      ```

      Sets the fallback policy.

      Parameters:
      :   `fallbackPolicy` - the new fallback policy


- Overview
- Package
- Class
- Use
- Tree
- Deprecated
- Index
- Help

- Prev Class
- Next Class

- Frames
- No Frames

- All Classes

- Summary:
- Nested |
- Field |
- Constr |
- Method

- Detail:
- Field |
- Constr |
- Method
